# Invasive pneumococcal disease rates linked to meteorological factors and respiratory virus circulation (Catalonia, 2006–2012)

**DOI:** 10.1186/s12889-016-3061-6

**Published:** 2016-05-13

**Authors:** Pilar Ciruela, Sonia Broner, Conchita Izquierdo, Sergi Hernández, Carmen Muñoz-Almagro, Roman Pallarés, Mireia Jané, Angela Domínguez, on behalf of the Working Group of Invasive Pneumococcal Disease of Catalonia

**Affiliations:** Agència de Salut Pública de Catalunya. Generalitat de Catalunya, Roc Boronat 81-95, 08005 Barcelona, Spain; Hospital Universitario Sant Joan de Deu, P° Sant Joan de Deu 2, 08950 Esplugues, Barcelona, Spain; Hospital Universitario Bellvitge, Feixa Llarga s/n, 08907 L’Hospitalet, Barcelona, Spain; Universidad de Barcelona, Casanova 143, 08036 Barcelona, Spain; CIBER de Epidemiología y Salud Pública (CIBERESP), Instituto de Salud Carlos III, Monforte de Lemos, 3-5, 28029 Madrid, Spain

**Keywords:** *Streptococcus pneumoniae*, IPD, Respiratory viruses, Influenza, Meteorological variables

## Abstract

**Background:**

To study the impact of meteorological data and respiratory viral infections on invasive pneumococcal disease (IPD) rates.

**Methods:**

We analysed all notifications of IPD and respiratory viral infections to the Microbiological Reporting System of Catalonia (2006–2012). Correlations between rates of IPD and viral infections (influenza virus, respiratory syncytial virus [RSV] and adenovirus), and meteorological variables (temperature, humidity, hours of sunshine, wind speed and number of days with rainfall) were assessed using Spearman’s correlation coefficient and negative binomial regression models.

**Results:**

We found significant correlations between monthly rates of IPD and monthly rates of all respiratory viruses and meteorological factors.

However, after multiple regression analysis, associations remained between IPD rates and influenza rates and reductions in temperature in the total population, and between IPD rates and adenovirus rates in children aged <5 years.

When models were repeated for the total population using data from the preceding month, IPD rates increased when RSV was circulating and when the temperature was lower. In children aged <5 years, RSV circulation was associated with increased IPD rates.

**Conclusions:**

IPD rates were linked to increased activity of some respiratory viruses and reductions in temperature. Preventive measures, including influenza vaccination, may help reduce IPD.

**Electronic supplementary material:**

The online version of this article (doi:10.1186/s12889-016-3061-6) contains supplementary material, which is available to authorized users.

## Background

Invasive pneumococcal disease (IPD) remains a serious public health problem. The epidemiology of the disease shows seasonal patterns with a higher incidence in winter months [1, 2] related to increased respiratory virus activity [3–5].

Host, sociodemographic, and climatic factors, and previous respiratory viral infections [2, 6–8] have been associated with increases in IPD.

Viral respiratory infections, particularly those caused by the influenza virus and respiratory syncytial virus (RSV) enhance the incidence of IPD using different mechanisms [9]. Viral respiratory infections damage respiratory epithelial cells [10] and predispose to secondary bacterial infections by promoting bacterial adhesion to the respiratory epithelium [9, 11]. Moreover, viral infection may reduce cell clearance of *Streptococcus pneumoniae* in previously-colonized respiratory cells [12], easing the spread by aspiration or adhesion to sterile areas.

In Catalonia, Spain, the incidence of viral respiratory infection [5] and IPD [13] is high, although a reduction in IPD rates has been detected in recent years, probably influenced by the administration of pneumococcal vaccines. The 23-valent pneumococcal polysaccharide vaccine (PPV23) and the influenza vaccine are recommended for persons with high-risk medical conditions and for all persons aged >60 years [14]. The 7-valent pneumococcal conjugate vaccine (PCV7) and 13-valent pneumococcal conjugate vaccine (PCV13) are not currently included in the routine vaccination schedule of Catalonia, and are only indicated and offered for free in children aged <5 years with risk factors [14]. Estimated vaccine coverage are around 50 % in adults for the PPV23 [15] and 55.1, 1.4 and 12.5 % for the PCV13, PCV10 and PCV7, respectively, in children [16].

Several authors have studied the association between IPD and respiratory virus infection and meteorological variables and although most found an association between IPD and influenza virus and RSV [2, 17–19], the results differ substantially according to the statistical methods used [8, 20].

The objective of this study was to investigate the correlation between fluctuations in respiratory virus activity and meteorological data with IPD rates in Catalonia over a 7-year period.

## Methods

### Setting

Catalonia is a region in the northeast of Spain located at latitude 41.6°N. It has a temperate maritime climate with winters during December-March. The total population in 2012 was 7,570,908, of whom 423,308 were aged <5 years, 5,860,079 aged 5–64 years, and 1,287,521 aged ≥65 years [21].

### Study design

An ecological study was carried out in Catalonia from January 2006 to December 2012. All prospective notifications of IPD and adenovirus, influenza virus, and RSV infections reported to the Microbiological Reporting System of Catalonia (MRSC) coordinated by the Public Health Agency of Catalonia were analyzed.

The MRSC has been a sentinel enhanced surveillance system [22] since 1995 and involves 50 health care centers representing over 82.2 % of hospital beds in public hospitals in Catalonia (45 public hospitals acute out of a total of 67) [23]. Microbiologists report acute cases of infectious diseases, with sociodemographic and clinical information, case by case or weekly.

### Variables

IPD was defined as *S. pneumoniae* isolation or detection of nucleic acids by polymerase chain reaction (PCR) or antigen detection in normally sterile body fluids. Respiratory virus infections were diagnosed by culture, PCR, or detection of antigen from clinical respiratory specimens.

The data collected included the number of cases of IPD (dependent variable) and respiratory virus infections (independent variable) per month in the study period. To calculate incidence rates, the population for each year was obtained from the Statistical Institute of Catalonia [21].

Meteorological data, including temperature, humidity, hours of sunshine, wind speed and number of days with rainfall were obtained from the Spanish National Statistics Institute and the Meteorological Service of Catalonia [24, 25]. Data were obtained from the four provincial meteorological stations in Catalonia (Barcelona, Girona, Lleida and Tarragona). Temperature (°C), hours of sunshine, wind speed (km/h), relative humidity (%) and number of days with rainfall was recorded each month (see Additional file 1).

### Statistical methods

Age-specific incidence rate ratios (IRR) with 95 % confidence intervals (CI) were calculated for IPD, and influenza virus, RSV and adenovirus infections.

The relationships between monthly rates of IPD and rates of respiratory virus infection and monthly means of meteorological variables were assessed using Spearman’s rank correlation coefficient. The analysis was repeated with data on viral infections and meteorological variables from the previous month (1-month lag) to analyze their influence on the rates of IPD [8].

Regression analysis was used to further assess the relationship between monthly rates of IPD, viral infections and meteorological variables. Only variables that were significant in the correlation analysis were included. Negative binomial regression models were constructed for the total population and for the <5 years, 5–64 years, and ≥65 years age groups.

In all models, the monthly rate of IPD was the outcome variable. The explanatory variables (all binary) used were the monthly rate of viral infections, mean average monthly temperature >17 °C, mean average monthly hours of sunshine >7 h, mean average monthly wind speed >11 km/h, mean average monthly humidity >64 %, and mean average monthly number of days with rainfall >7 days. Viruses were considered as circulating during a given month when the viral infection was reported from at least two people during that month [8]. Correlations with meteorological variables were measured. The analyses were performed using the Statistical Package for Social Sciences (SPSS 19.0 for Windows) and R 2.13.0 (R Development Core Team 2014).

## Results

### Invasive pneumococcal disease

During the study period there were 8044 episodes of IPD and the incidence rate (IR) was 15.5 per 100,000 persons-year. Most cases were diagnosed by culture (94.7 %), and 4664 (58 %) were male. The median age of cases was 55 (range 0–100 years) and the highest incidence rate was in children aged <5 years (IR: 50 per 100,000 persons-year) (Table 1). Pneumonia was the most frequent clinical presentation with 5945 cases (73.9 %), of which pneumonia with empyema represented 11.7 % (693 cases), followed by non-focal bacteremia (1347 cases; 16.7 %), and meningitis (606 cases; 7.5 %). Other clinical presentations of IPD represented 1.8 % (146) cases, including peritonitis (86 cases; 58.9 %), arthritis (43 cases; 29.5 %), cellulitis (6 cases; 4.1 %), cholecystitis (3 cases, 2.1 %), pericarditis (3 cases, 2.1 %), endocarditis (2, 1.4 %), mastoiditis (2, 1.4 %) and endophthalmitis (1, 0.7 %). A total of 74.3 % of strains (5973) were serotyped: the most frequent serotypes were 1 (931; 15.6 %), 19A (596; 10.0 %), 7 F (528; 8.8 %), 3 (523; 8.8 %) and 14 (379; 6.3 %).

**Table 1:** Incidence rates of invasive pneumococcal disease and respiratory virus infection by age group. Catalonia, 2006–2012

	All ages		<5 years		5–64 years		≥65 years	
	n	Incidence rate^a^	n	Incidence rate^a^	n	Incidence rate^a^	n	Incidence rate^a^
IPD	8044	15.5 (15.2–15.9)	1430	50.0 (47.5–52.7)	3528	8.7 (8.5–9.0)	3020	35.3 (34.0–36.5)
Influenza	6845	13.2 (12.9–13.5)	2588	90.5 (87.1–94.1)	3710	9.2 (8.9–9.5)	472	5.5 (5.0–6.0)
RSV	9507	18.4 (18.0–18.7)	9079	317.5 (311.2–324.1)	283	0.7 (0.6–0.8)	81	1.0 (0.8–1.2)
Adenovirus	1211	2.3 (2.2–2.5)	991	34.7 (32.5–36.9)	181	0.5 (0.4–0.5)	32	0.4 (0.3–0.5)

*Abbreviations*: *IPD* invasive pneumococcal disease, *RSV* respiratory syncytial virus

^a^per 100,000 persons-year

### Respiratory virus infection

During the study period, 17,563 respiratory virus infections were reported. The highest incidence rate was in children aged <5 years (Table 1). Of the 9507 cases of RSV (IR: 18.4 per 100,000 persons-year), 9,079 (IR: 317.5 per 100,000 persons-year) occurred in children aged <5 years. There were 6,845 cases of influenza virus infection (IR: 13.2 per 100,000 persons-year) of which 2,588 were in children aged <5 years (IR: 90.5 per 100,000 persons-year) and almost 90 % were influenza A (5,922). Of the 1,211 cases of adenovirus infection (IR: 2.3 per 100,000 persons-year) 991 cases occurred in children aged <5 years (IR: 34.7 per 100,000 persons-year).

### Seasonal distribution

**Fig. 1:**
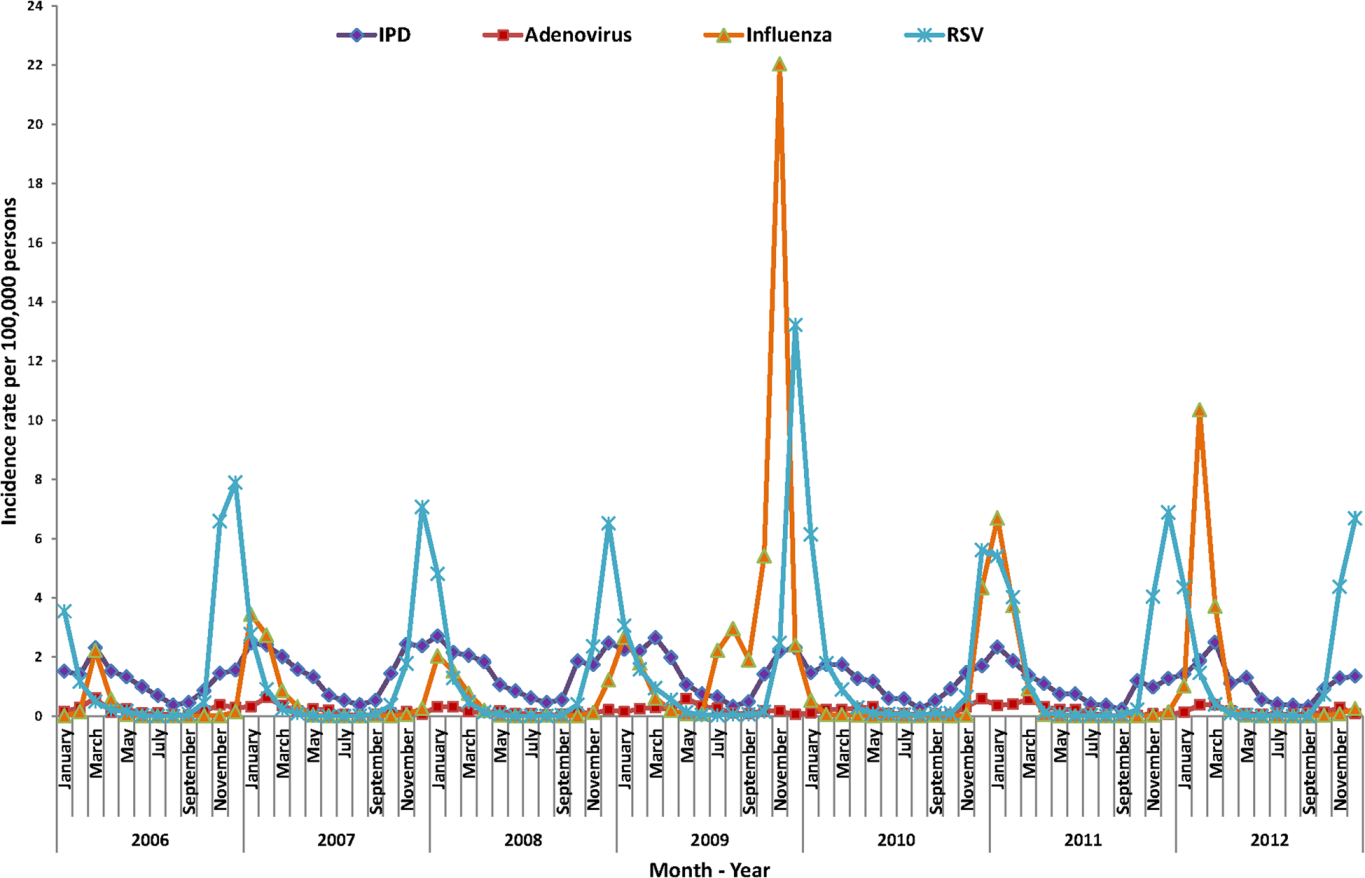
Monthly rates of invasive pneumococcal disease and respiratory virus detection over the 7-year study period. Catalonia, 2006–2012

IPD showed a seasonal pattern, with the highest incidence in the winter months (November to March), coinciding with the peak of influenza activity (January to March). RSV infections started to rise 4 to 6 weeks before the peaks of IPD and influenza activity. In 2009, there was a sharp increase in influenza virus infections due to the global H1N1 influenza pandemic 4 weeks before the increase in RSV incidence (Fig. 1). The incidence of adenovirus was low, with an increase in February and March.

### Meteorological factors

During the study period the mean and median temperature were 16.5 °C and 16.7 °C (standard deviation: 6.4 °C; range: 6.8 °C–27.6 °C), the mean and median hours of sunshine were 6.9 h (standard deviation: 2.0 h; range: 3.3 h–10.7 h), the mean and median wind speed were 10.6 Km/h and 10.8 Km/h (standard deviation: 1.4 Km/h; range: 7.3 Km/h-14.0 Km/h), the mean and median relative humidity were 63.3 % and 62.4 % (standard deviation: 6.8 %; range: 49.8 %–80.56 %) and the mean and median number of days with rainfall were 7 days and 6 days (standard deviation: 3 days; range: 2 days–15 days).

**Fig. 2:**
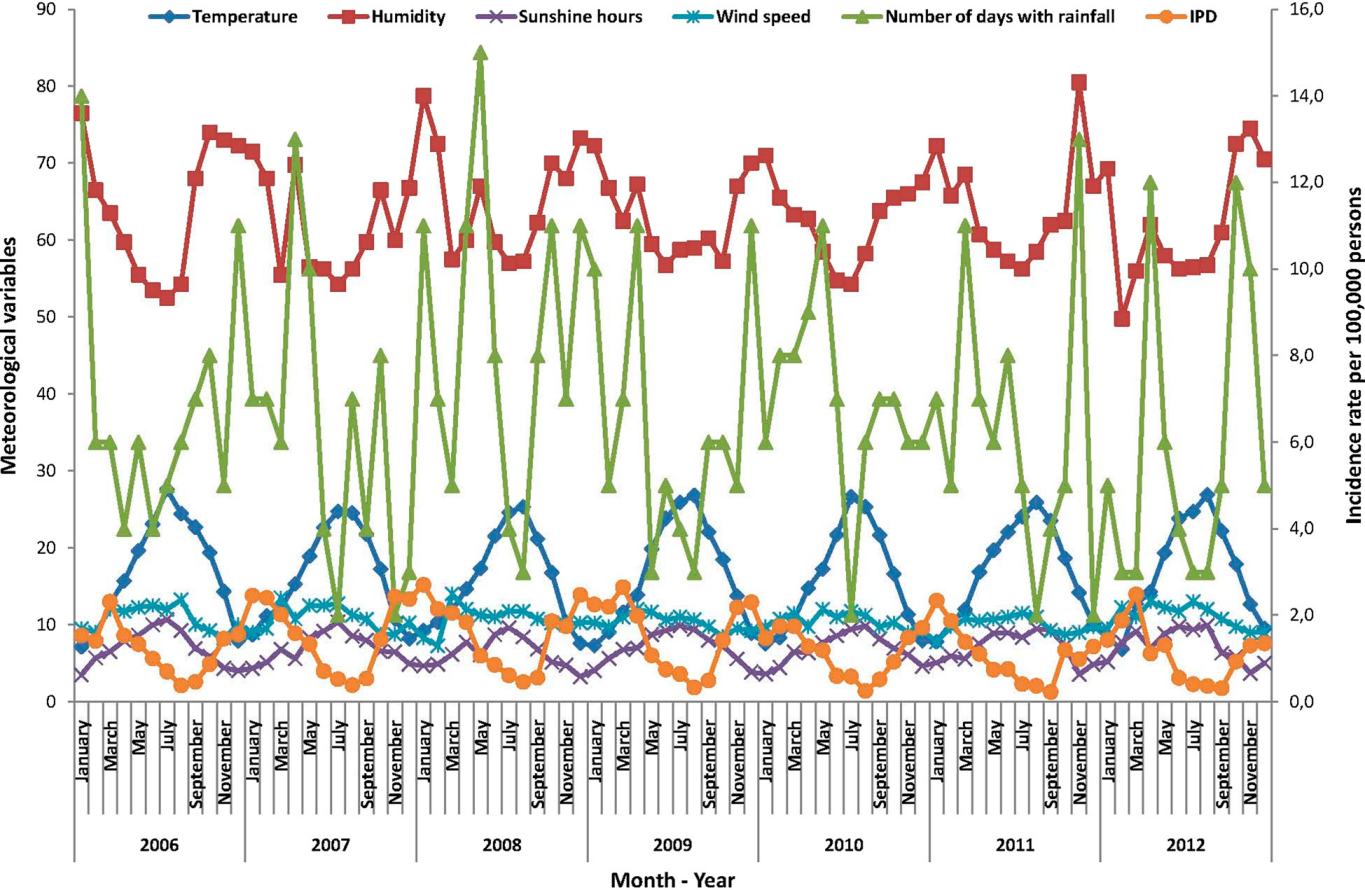
Monthly rates of invasive pneumococcal disease and monthly meteorological data over the 7-year study period. Catalonia, 2006–2012

Figure 2 shows the monthly variations in meteorological variables for the study period compared with the monthly rate of IPD. There was an increase in IPD when the temperature, hours of sunshine and wind speed were lower and humidity and number of days with rainfall was higher.

### Correlation between IPD, respiratory virus infection, and meteorological factors

There was a correlation between monthly detection rates of all respiratory viral infections and meteorological variables with rates of IPD (Table 2). These results remained when the analysis was repeated with a 1-month lag. The correlation between RSV and influenza virus and IPD remained after stratifying the data by all age groups, in the concurrent month and with a 1-month lag. Adenovirus infections correlated with IPD in the <5 years age group (in the concurrent month and with a 1-month lag) and in the 5–64 years age group only in the concurrent month. All meteorological variables correlated with IPD in the concurrent month and with a 1-month lag.

**Table 2:** Correlation of monthly rates of invasive pneumococcal disease with rates of respiratory virus infection in concurrent and preceding month. Catalonia, 2006–2012

Variable	All ages		<5 years		5–64 years		≥65 years	
	No lag ^a^ (*p*-value)	1-month lag ^b^ (*p*-value)	No lag^a^ (*p*-value)	1-month lag^b^ (*p*-value)	No lag^a^ (*p*-value)	1-month lag^b^ (*p*-value)	No lag^a^ (*p*-value)	1-month lag^b^ (*p*-value)
Influenza	0.71 (<0.001)	0.64 (<0.001)	0.60 (<0.001)	0.57 (<0.001)	0.65 (<0.001)	0.57 (<0.001)	0.41 (<0.001)	0.36 (0.001)
RSV	0.77 (<0.001)	0.80 (<0.001)	0.63 (<0.001)	0.61 (<0.001)	0.50 (<0.001)	0.44 (<0.001)	0.38 (<0.001)	0.38 (<0.001)
Adenovirus	0.61 (<0.001)	0.39 (<0.001)	0.55 (<0.001)	0.32 (0.003)	0.23 (0.040)	0.17 (0.137)	0.09 (0.396)	−0.05 (0.663)
Temperature	−0.85 (<0.001)	−0.84 (<0.001)	−0.65 (<0.001)	−0.64 (<0.001)	−0.82 (<0.001)	−0.82 (<0.001)	−0.87 (<0.001)	−0.86 (<0.001)
Humidity	0.48 (<0.001)	0.62 (<0.001)	0.39 (<0.001)	0.56 (<0.001)	0.46 (<0.001)	0.60 (<0.001)	0.48 (<0.001)	0.63 (<0.001)
Sunshine hours	−0.67 (<0.001)	−0.79 (<0.001)	−0.53 (<0.001)	−0.62 (<0.001)	−0.65 (<0.001)	−0.76 (<0.001)	−0.67 (<0.001)	−0.81 (<0.001)
Wind speed	−0.31 (0.005)	−0.58 (<0.001)	−0.29 (0.008)	−0.54 (<0.001)	−0.30 (0.006)	−0.57 (<0.001)	−0.28 (0.010)	−0.54 (<0.001)
Days with rainfall	0.29 (0.008)	0.25 (0.021)	0.25 (0.024)	0.24 (0.031)	0.30 (0.006)	0.25 (0.022)	0.27 (0.013)	0.27 (0.013)

*Abbreviations*: *RSV* respiratory syncytial virus

*Note*: Numbers are Spearman’s rank correlation coefficients

^a^Concurrent month

^b^Preceding month

### Regression models

The results of the bivariate regression analysis presented in Table 3 showed significant associations between adenovirus, influenza virus and RSV and IPD. After stratifying by age groups, the association remained significant in all age groups for influenza virus and RSV. Adenovirus was only significant in children aged <5 years.

**Table 3:** Results of bivariate regression analysis between invasive pneumococcal disease rates and respiratory virus infection rates and temperature for concurrent months and 1-month lag. Catalonia, 2006–2012

Variable	All ages		<5 years		5–64 years		≥65 years	
	No lag^a^	1-month lag^b^	No lag^a^	1-month lag^b^	No lag^a^	1-month lag^b^	No lag^a^	1-month lag^b^
	IRR (95 % CI)	IRR (95 % CI)	IRR (95 % CI)	IRR (95 % CI)	IRR (95 % CI)	IRR (95 % CI)	IRR (95 % CI)	IRR (95 % CI)
Influenza	2.13 (1.71–2.63)	1.85 (1.46–2.33)	1.86 (1.44–2.39)	1.59 (1.22–2.07)	2.08 (1.69–2.57)	1.84 (1.47–2.31)	1.37 (1.03–1.83)	1.29 (0.96–1.74)
RSV	2.41 (1.78–3.22)	2.94 (2.21–3.87)	2.49 (1.76–3.48)	3.22 (2.31–4.47)	1.82 (1.45–2.29)	1.76 (1.40–2.24)	1.59 (1.13–2.30)	1.66 (1.19–2.40)
Adenovirus	3.47 (1.39–7.30)	3.44 (1.37–7.25)	4.43 (2.25–8.61)	1.62 (0.85–2.89)	1.14 (0.88–1.47)	1.11 (0.86–1.44)	1.35 (0.85–2.30)	1.19 (0.74–2.03)
Temperature >17 °C	0.39 (0.35–0.46)	0.45 (0.37–0.55)	0.44 (0.35–0.55)	0.55 (0.43–0.71)	0.39 (0.33–0.46)	0.45 (0.37–0.55)	0.37 (0.32–0.44)	0.41 (0.34–0.49)

*Abbreviations*: *IRR* Incidence rate ratio, *CI* Confidence interval, *RSV* respiratory syncytial virus

^a^Concurrent month

^b^Preceding month

The same results were obtained in the total population when the models were repeated using a 1-month lag. RSV was significant in all age groups and influenza virus in children aged <5 years and persons aged 5–64 years. Adenovirus was not significant in any age group.

The mean monthly temperature correlated closely with mean monthly hours of sunshine (*r* = 0.85, *p* < 0.001), mean monthly wind speed (*r* = 0.44, *p* < 0.001), mean monthly relative humidity (*r* = −0.64, *p* < 0.001) and mean monthly number of days with rainfall (*r* = −0.34, *p* = 0.001) and therefore we only included temperature in the regression models. In addition, temperature had shown the closest correlation with IPD.

The results of the multivariate regression analyses for all age groups are presented in Table 4. Accounting for the effects of independent variables in all age groups, rates of IPD increased during the circulation of the influenza virus (IRR: 1.26, 95 % CI: 1.03–1.54) and decreased when the monthly temperature was >17 °C (IRR: 0.47, 95 % CI: 0.39–0.57). When the models were repeated using data from the preceding month, rates of IPD increased when the RSV was circulating (IRR: 1.81, 95 % CI: 1.36–2.41) and decreased when the monthly temperature was >17 °C (IRR: 0.56, 95 % CI: 0.45–0.70).

**Table 4:** Results of multivariate regression analysis of the relationship between invasive pneumococcal disease rates and respiratory virus infection rates and temperature for concurrent months and 1-month lag. Catalonia, 2006–2012

Variable	All ages		<5 years		5–64 years		≥65 years	
	No lag^a^	1-month lag^b^	No lag^a^	1-month lag^b^	No lag^a^	1-month lag^b^	No lag^a^	1-month lag^b^
	IRR (95 % CI)	IRR (95 % CI)	IRR (95 % CI)	IRR (95 % CI)	IRR (95 % CI)	IRR (95 % CI)	IRR (95 % CI)	IRR (95 % CI)
Influenza	1.26 (1.03–1.54)	1.09 (0.87–1.36)	1.16 (0.90–1.50)	1.06 (0.80–1.42)	1.22 (0.98–1.53)	1.20 (0.89–1.34)	0.92 (0.76–1.14)	0.94 (0.74–1.21)
RSV	1.15 (0.89–1.48)	1.81 (1.36–2.41)	1.41 (1.00–1.97)	2.57 (1.78–3.71)	1.12 (0.91–1.37)	1.15 (0.90–1.48)	1.04 (0.81–1.33)	1.13 (0.85–1.52)
Adenovirus	1.58 (0.88–2.74)	1.32 (0.68–2.42)	2.47 (1.38–4.53)	1.00 (0.59–1.68)	1.14 (0.96–1.35)	1.10 (0.90–1.34)	1.32 (0.96–1.83)	1.19 (0.82–1.77)
Temperature >17 °C	0.47 (0.39–0.57)	0.56 (0.45–0.70)	0.58 (0.44–0.77)	0.76 (0.56–1.02)	0.47 (0.37–0.59)	0.54 (0.41–0.70)	0.37 (0.31–0.44)	0.42 (0.34–0.51)

*Abbreviations*: *IRR* Incidence rate ratio, *CI* Confidence interval *RSV* respiratory syncytial virus

^a^Concurrent month

^b^Preceding month

The increase in the rate of IPD was significant during the adenovirus circulation season in children aged <5 years (IRR: 2.47, 95 % CI: 1.38–4.53). Rates of IPD decreased during months with a monthly temperature >17 °C in all age groups (<5 years: IRR: 0.58, 95 % CI: 0.44–0.77; 5–64 years: IRR: 0.47, 95 % CI: 0.37–0.59 and ≥65 years: IRR: 0.37, 95 % CI: 0.31–0.44). When the models were repeated using a 1-month lag, rates of IPD increased during RSV circulation (IRR: 2.57, 95 % CI: 1.78–3.71), in the <5 years age group. Rates of IPD decreased when the monthly temperature was >17 °C in the 5–64 years age group (IRR: 0.54, 95 % CI: 0.41–0.70) and ≥65 years age group (IRR: 0.42, 95 % CI: 0.34–0.51).

The results of the models were similar when the analyses were repeated excluding data on influenza virus infection during the 2009 season, which appeared to be outliers due to the influenza A H1N1 pandemic.

## Discussion

The results of this study showed a temporal association between high IPD rates and increased respiratory viral activity according to age groups and an association between increases in IPD incidence and reductions in temperature.

In the univariate model, there were associations between IPD and all respiratory virus infections studied (influenza virus, RSV, and adenovirus), in the whole population and in most age groups in the concurrent month and in the previous month. Kim et al. [2] found similar results in adults but not in children, showing an association between IPD and respiratory virus infections (influenza, RSV and adenovirus) in children that was weaker and less immediate than in adults, with a 4-week lag period. The study by Ampofo et al. [18] in children aged <18 years found an association between IPD and influenza and RSV infection in all periods studied (concurrent month and four previous weeks) although, unlike our study, they only found association between IPD and adenovirus in the two previous weeks between IPD and adenovirus. Other authors have found an association between IPD and RSV but no association between IPD and adenovirus [26, 27] and influenza infection [26–28] in children.

In the multivariate model, a high incidence of IPD was associated with an increase in influenza virus infection in the concurrent month and an increase in RSV infection in the previous month in the total population. In children aged <5 years there was an association between IPD and adenovirus in the concurrent month and with RSV in the previous month. No association was found in other age groups. A reduction in temperature was associated with high IPD incidence in all age groups.

The association between IPD and influenza virus infection in adults has been described by various authors using different statistical models. A multivariate New Zealand study by Murdoch et al. [8] that included respiratory virus infections (influenza, RSV, adenovirus and parainfluenza virus) and meteorological factors found an association between IPD and influenza virus infection in the whole population and in adults aged ≥65 years. A UK study by Nicoli et al. [20] found similar results in a regression model including only influenza virus infection and RSV when they adjusted by temperature. In contrast, our results showed an association between IPD and influenza virus infection in the whole population but not in older age groups. This was probably due to the small number of influenza virus infection cases reported in adults aged ≥65 years. The increase in reported influenza infections from 2009 onwards, after the H1N1 pandemic, was probably due to increased physician awareness of the disease and increased reporting, leading to more confirmed diagnoses.

The association between IPD and RSV found in our study has, with some differences, been described by Nicoli et al. [20], who found an association in all age groups in the concurrent month and the previous month: our results show only an association in the total population and in children aged <5 years, and only in the previous month. Other studies found no association in any age group or at any time [8].

Few multivariate studies have included adenovirus in the analysis. Murdoch et al. [8] found an association between IPD and adenovirus infection in the whole population and in adults aged ≥65 years in the previous month. In contrast, we observed an association in children aged <5 years in the concurrent month but not in other age groups. The difference may be explained, in part, by the small number of cases in these age groups.

As stated previously, the meteorological variables studied (hours of sunshine, wind speed, relative humidity and number of days with rainfall) were collinear with temperature and therefore we only included temperature in the regression models. The results showed that a lower temperature was associated with IPD rates in all age groups in the concurrent month and in the previous month, except in children aged <5 years, where it was only significant in the concurrent month. A decrease in temperature might be linked with IPD because it could increase host susceptibility to pneumococcal infection [20, 28].

Other studies have included other meteorological variables. Kim et al. [2] found an association between IPD and temperature in all age groups but an association between IPD and air pollution (SO^2^) only in adults. Murdoch et al. [8] included pollution, rainfall, humidity, hours of sunshine and temperature in a regression model and found pollution was associated with IPD in the 5–65 years age group in the concurrent month and in adults aged >65 years in the previous month, and humidity was associated with IPD in children aged <5 years in the preceding month. They found no association between reduced temperatures and IPD in any age group, in contrast to our results and those of Nicoli et al. [20].

The main strength of this study is that the data came from the MRSC and were confirmed and completed by using other sources such as the Spanish and Catalan IPD reference centers. The same source was used for all microorganisms studied, and hospital and community cases were included. The sample size of the study is another strength.

The study has some limitations. We did not include other viruses (rhinovirus or metapneumovirus) because they were not reported to the MRSC. The parainfluenza virus was also excluded because cases were not reported regularly by hospitals and this could have biased the results. Although only a small number of influenza virus infections were reported in adults aged ≥65 years, we detected an increase after 2008, probably due to more reports and diagnoses after the influenza pandemic. Likewise, more significant associations might have been found if the study period had been longer.

## Conclusions

Our results showed that temperature and seasonality were factors influencing influenza virus and RSV activity. In the whole population, both viral infections were associated with high IPD rates, while in children aged <5 years an association was found between adenovirus and RSV infections and IPD. Our results reinforce the idea that influenza vaccination may help prevent IPD.

## Availability of data and material

The dataset supporting the conclusions of this article is included within the article.

## Additional file

Additional file 1: Distribution of IPD, respiratory viruses and meteorological factors by month and year. Catalonia, 2006–2012. (XLS 46 kb)

## Abbreviations

CI: confidence intervals
Idescat: Statistical Institute of Catalonia
IPD: invasive pneumococcal disease
IR: incidence rate.
IRR: incidence rate ratios
MRSC: Microbiological Reporting System of Catalonia
PCR: polymerase chain reaction
PCV13: 13-valent pneumococcal conjugate vaccine
PCV7: 7-valent pneumococcal conjugate vaccine
PPV23: 23-valent pneumococcal polysaccharide vaccine
RSV: respiratory syncytial virus
